# Supplementary material for: High‐throughput, microscope‐based sorting to dissect cellular heterogeneity
Source: Mol Syst Biol. 2020 Jun 5;16(6):e9442. doi: 10.15252/msb.20209442 (PMC7273721; doi:10.15252/msb.20209442)
Supplement: Supplementary file 1 — Appendix [file MSB-16-e9442-s001.pdf]

## Table of Contents


Table of Contents ........ 1

Extended description of general reagents, DNA oligonucleotides and plasmids. ........ 2

Extended description of the construction of site-saturation mutagenesis library for the SV40 NLS ........ 3

Extended description of lentivirus production ........ 4

Extended description of the creation of clonal cell lines ........ 4

Extended description of the recombination of single-variant SV40 NLS clones or the library into the U-2 OS-landing pad line expressing H3-Dendra2 ........ 4

Extended description of the Metamorph journals used for imaging, analysis, and photoactivation ........ 5

Extended description of validation of single NLS variants ........ 5

Extended description of the Visual Cell Sorting on cells treated with paclitaxel ........ 6

Extended description of the differentially expressed genes analysis ........ 6

Appendix Supplementary Figures ........ 9

## Extended description of general reagents, DNA oligonucleotides and plasmids.

To create attB-H3-Dendra2, the Dendra2 open reading frame was obtained from Dendra2-Lifeact7 (a gift from Michael Davidson Addgene #54694) and cloned downstream of the H3 open reading frame from mEmerald-H3-23 (a gift from Michael Davidson Addgene #54115) and into the backbone of attB-EGFP-PTEN-IRES-mCherry[53].

To create attB-H3-Dendra2-P2A-H2B-miRFP703, attB-H3-Dendra2 and pH2B-miRFP703 (a gift from Vladislav Verkhusha, Addgene #80001) were combined and a P2A sequence included in the Gibson overhang regions between Dendra2 and miRFP.

To create pLenti-CMV-H3-Dendra2, the H3-Dendra2 reading frame in attB-H3-Dendra2 replaced the open reading frame in pLenti CMV rtTA3 Blast (w756-1), a gift from Eric Campeau (Addgene #26429).

To create attB-Nterm-CMPK-miRFP (the destination vector for the NLS library), a gBlock encoding an EcoRI site in-frame and upstream of CMPK (IDT; based off a previously published SV40 NLS construct[21]) was combined with the miRFP open reading frame from pH2B-miRFP703 and inserted into the backbone of attB-H3-Dendra2-P2A-H2B-miRFP703.

To create attB-NLS-CMPK-miRFP and all single, double, and triple amino acid variants, the attB-Nterm-CMPK-miRFP vector was digested with EcoRI for 2 hours at 37 C. Then, the digested plasmid and an oligo that contained the NLS (wild-type or variant of interest) and 55 C overhangs complementary to the edges of the cut site were incubated in a Gibson reaction in a one to three molar ratio and transformed, as per manufacturer's instructions.

To create pLenti-CMV-NLS-Dendra2x3-P2A-H2B-miRFP, three PCRs of Dendra2 (template derived from Dendra2-Lifeact7) were performed: one with an N-terminal NLS appended on the forward primer and a Gly-rich linker on the reverse; one with the Gly-rich linker on the forward primer and a second, non-identical Gly-rich linker on the reverse primer; and one with the

second Gly-rich linker on the forward primer and a stop codon on the reverse primer. These were combined with an attB construct backbone[53] to create attB-NLS-Dendra2x3. In a second cloning step, H2B-miRFP from pH2B-miRFP703 was appended downstream to create attB-NLS-Dendra2x3-P2A-miRFP. Finally, the Dendra2x3-P2A-H2B-miRFP open reading frame was cloned into pLenti CMV rtTA3 Blast (w756-1).

To create pLenti-CMV-mBeRFP-NLS, a gBlock encoding codon-optimized mBeRFP[48] (IDT) was cloned into pLenti CMV rtTA3 Blast (w756-1) with an NES encoded into Gibson overhangs.

## Extended description of the construction of site-saturation mutagenesis library for the SV40 NLS

The library of all possible SV40 NLS missense variants was constructed using a Gibson cloning approach. Eleven primer pairs – 1 for each NLS codon, plus 2 codons upstream and 2 codons downstream of the NLS – were designed (The Reagents and Tools Table). For each pair, the forward primer contained a 3' annealing region (Tm ~55C), an NNK codon, and a 5' Gibson homology region (Tm ~55C). The reverse primer comprised of the reverse complement of the forward primer Gibson homology region. Each primer pair was used in a separate PCR reaction that included attB-NLS-CMPK-miRFP as the template, and 5ul of each reaction were run on a 1% gel to check for product. The remaining 20ul was DpnI digested for 2 hours at 37 C to remove template plasmid, cleaned using DNA Clean & Concentrator-5 (Zymo Research D4013), subject to a 1-piece Gibson reaction, and transformed into chemically competent *E. coli*. Bulk transformant cultures were grown overnight and harvested using GenElute HP Plasmid DNA Midiprep Kit (Sigma, NA0200-1KT). DNA preps containing single codon variant were subsequently mixed such that each prep contributed an equal amount of DNA. The final library contained 346 NNK nucleotide variants which, due to codon degeneracy in the genetic code, encode for 209 single amino acid variants.

## Extended description of lentivirus production

To produce lentivirus, HEK293T cells were plated in clear plastic 6 well plates (VWR, cat. no. 10062-892) at 4.5e5 cells per well. The next day, cells in each well were transfected with 1,125 ng psPAX2 (a gift from Didier Trono, AddGene #12260), 375 ng pMD2.G (a gift from Didier Trono, AddGene #12259), and 1,500ng of pLenti transfer vector using 6ul of FuGENE6 (Promega, cat. no. E2691) according to manufacturer's instructions. Media was replaced 24 hours after transfection and collected at 48 hours and 72 hours after transfection. Collected media was spun at 1000g for 5 minutes, then the viral supernatant was decanted and filtered using a 0.45um filter (VWR, cat. no. 28145-481). Finally, the virus was concentrated using PEG-it Virus Precipitation Solution (SBI, cat. no. LV810A-1) and stored at -80C.

## Extended description of the creation of clonal cell lines

To create the clonal U-2 OS landing pad and H3-Dendra2 expressing line, parental U-2 OS cells were transduced with lentivirus encoding the landing pad[53]and lentivirus encoding H3-Dendra2. Five days after transduction, BFP +ve / Dendra2 +ve cells were sorted using an Aria III (Pacific Blue and FITC Channels). Three days later, cells were sorted directly into 96 well plates containing 75ul of conditioned U-2 OS media. Every week, 50ul of normal media was added to the well. Wells were checked for surviving clones at 2 weeks and 3 weeks post-sorting.

To create the hTERT RPE-1 clonal line expressing NLS-Dendra2x3, mBeRFP-NES, and H2B-miRFP, lentiviruses encoding these constructs were added to the parental line, and single cell clones were similarly sorted and expanded in conditioned media in 96 well plates.

## Extended description of the recombination of single-variant SV40 NLS clones or the library into the U-2 OS-landing pad line expressing H3-Dendra2

To recombine NLS variants or the NLS library into cells, H3-Dendra2 expressing U-2 OS cells with the landing pad were subject to Lipofectamine 3000 (Thermo Fisher L3000015)

transfections in 6 well plates, T-25 flasks, or T-75 flasks, according to manufacturer instructions, with the following specifications: plated cells at 0.1e5 cells/well (24 well plate), 0.6e5 cells/well (6 well plate), 1.4e6 cells/flask (T-25), or 4.2e6/flask (T-75); transfected with 0.75ul/3.75ul/10.4ul/31.2ul Lipofectamine 3000, 1ul/5ul/13.9ul/41.7ul P3000 reagent, 500ng/2500ng/7000ng/21000ng total DNA at a by-weight ratio of 1/3 pCAG Bxb1 and 2/3 attB plasmid(s). Cells were transfected immediately after plating. Twenty-four hours after transfecti on, media was replaced. Doxycycline was added 48h after transfection. BFP negative, miRFP positive, Dendra2 positive cells were sorted 5-8 days after transfection.

## Extended description of the Metamorph journals used for imaging, analysis, and photoactivation

Visual Cell Sorting experiments have three Metamorph journals specified in the Metamorph high-throughput acquisition dialog box: a startup journal that initializes global variables accessed by other journals; an after-image journal that analyzes and activates cells; and an end of plate journal that turns off the laser. The microscope was directed to leave no overlap between images. In all experiments, nuclei touching the image border were removed. Site maps were customized by altering the htacquir.cfg configuration file. See the GitHub repository for the Metamorph journals and configuration files used.

## Extended description of validation of single NLS variants

Analysis of Metamorph-calculated nucleus and cytoplasm mean intensity values was done using Python (v3.6.5). To correct for differences in background intensity between wells and replicates, each image's miRFP background intensity was estimated using the 10$^{th}$ percentile of image pixel intensity values and this value was subtracted from each cell's mean nucleus and cytoplasmic miRFP intensity. Cells with no miRFP expression were removed with a gate that was determined by examination of the histogram of the mean miRFP intensity values for cells in

each well. An iPython notebook file with the code used to run the analysis is available on the GitHub repository.

### Extended description of the Visual Cell Sorting on cells treated with paclitaxel

To identify morphologically-normal and lobulated cells were imaged for unactivated Dendra2 (FITC channel; 100 ms). Then, a custom nuclear segmentation pipeline that optimizes detection of nuclear blebs, herniations, and other abnormalities was employed. First, a top hat filter with a maximum object area threshold of 5,000 pixels was applied to remove large autofluorescent objects, and a 3x3 low pass filter was applied to smooth nuclear fluorescence. To find nuclei, a flatten background filter (removal of objects < 20 pixels in size), Sobel edge detection kernel, and a sharpening kernel were used before applying Metamorph's "legacy heuristic" thresholding algorithm to create nuclear binaries. To clean the nuclear binaries, holes were filled; tunnels 1 pixel in width were filled in using a dilate function; holes were filled again; and then an erode function was used to reverse the enlarging effect of the dilate and edge detection steps. Finally, objects less than 20 pixels in size and greater than 400 pixels in size were discarded. Shape factors were computed for each remaining object. See the GitHub repository for the Metamorph journal that was used.

### Extended description of the differentially expressed genes analysis

We noted that the Visual Cell Sorting-derived lobulated and normal single cell RNA transcriptomes appeared to be confounded by a batch effect, despite the fact that cells were derived from a single well, sorted on the same day, and processed side by side (Experiment 1; Supplementary Figure 4). Using SoupX[28], which applies a linear PCA transformation that is determined by the RNA in empty 10X emulsion droplets, we found that cell-free RNA was responsible for this effect (Supplementary Figure 4B). To confirm this hypothesis, we repeated the experiment but sorted lobulated (800 ms activated) and morphologically normal (200 ms

activated cells) cells into the same bin (Experiment 2, “unseparated” population) and processed these cells in a single 10X lane. A UMAP embedding of the single cell transcriptomes derived from these unseparated cells showed a single cluster, confirming the batch effect in Experiment 1.

Although both SoupX and the mutual nearest neighbors algorithm[33] applied to cells in Experiments 1 corrected the batch effect (Supplementary Figure 4B), it is not statistically appropriate to use batch-corrected gene expression values to conduct a differentially-expressed gene test[33]. As such, we sought to use mutual nearest neighbors batch-corrected principle components to label the unseparated cells in Experiment 2 according to their similarity to the known lobulated or morphologically-normal cells in Experiment 1; and then use the raw gene expression values in Experiment 2 to conduct a differentially-expressed gene test. We noted that the first four principle components in the MNN batch correction output correlated with the visual phenotype (i.e. morphologically normal vs. lobulated) in Experiment 1. So, we performed on the cells in Experiment 1 a logistic regression to devise a score that distinguishes between morphologically normal and lobulated cells:

$$lobulation\ score \sim PC1 + PC2 + PC3 + PC4$$

This regression model was then applied to cells in Experiment 2 (unseparated cells, single 10X lane) and the model predictions, which we called the “lobulation scores”, were extracted for each cell. Using Moncole3[58,59], we performed on the Experiment 2 gene expression matrix a DEG test using the lobulation scores and Seurat-computed cell cycle scores as covariates:

$$Gene \sim lobulation\ score + S\ score + G2M\ score$$

By doing the differentially expressed gene test using Experiment 2, in which lobulated and normal cells were sequenced together, we avoided any batch-related artifacts. This operation is analogous to the cluster-based analysis originally discussed by Haghverdi and colleagues[33], but

uses a principle component-derived score rather than principle component-derived clusters as cell labels.

## Appendix Supplementary Figures

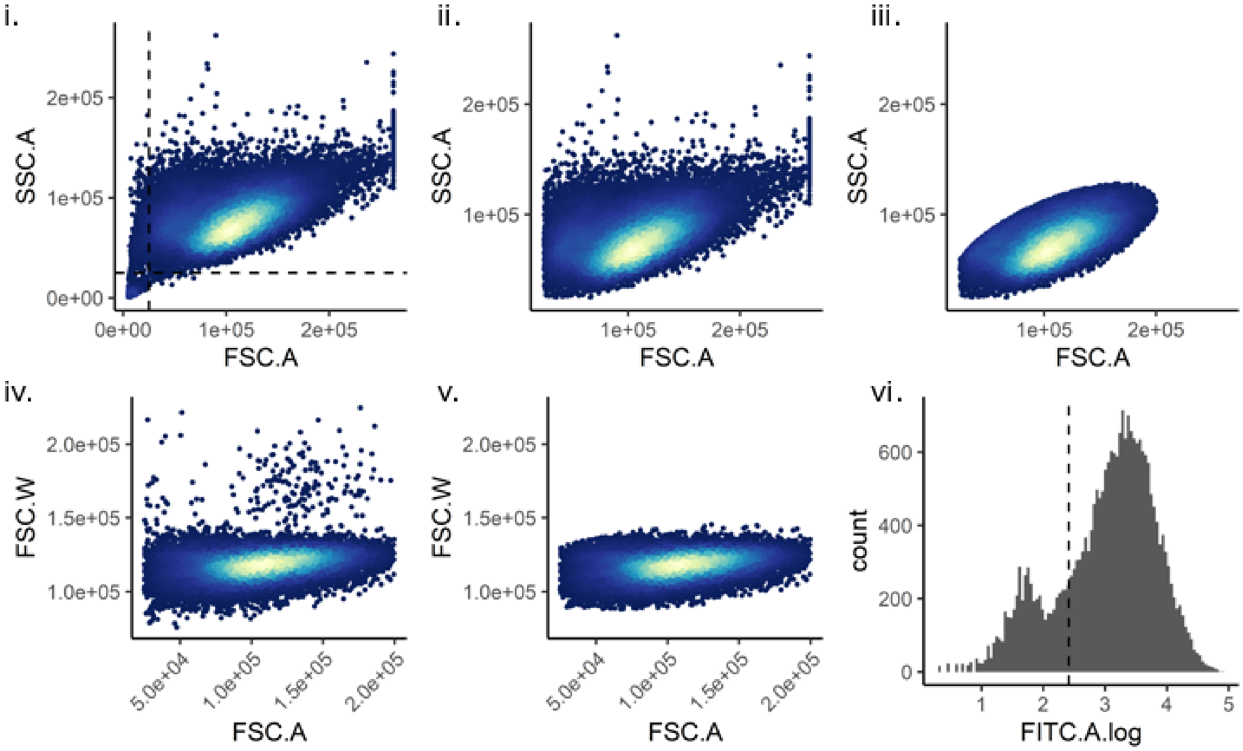


**Appendix Figure S1.** The gating scheme for selective photoactivation of cells expressing miRFP. Custom code using flowCore (v1.11.20) in R (v3.6.0) was used to gate the cells as follows. **(i)** Debris was removed using a SSC.A vs FSC.A plot. **(ii)** and **(iii)** A Mahalanobis distance filter was used to identify live cells on a SSC.A vs FSC.A plot. **(iv)** and **(v)** A Mahalanobis distance filter was used to identify single cells on a FSC.W vs FSC.A plot. **(vi)** Dendra2-positive cells were identified using a FITC plot.

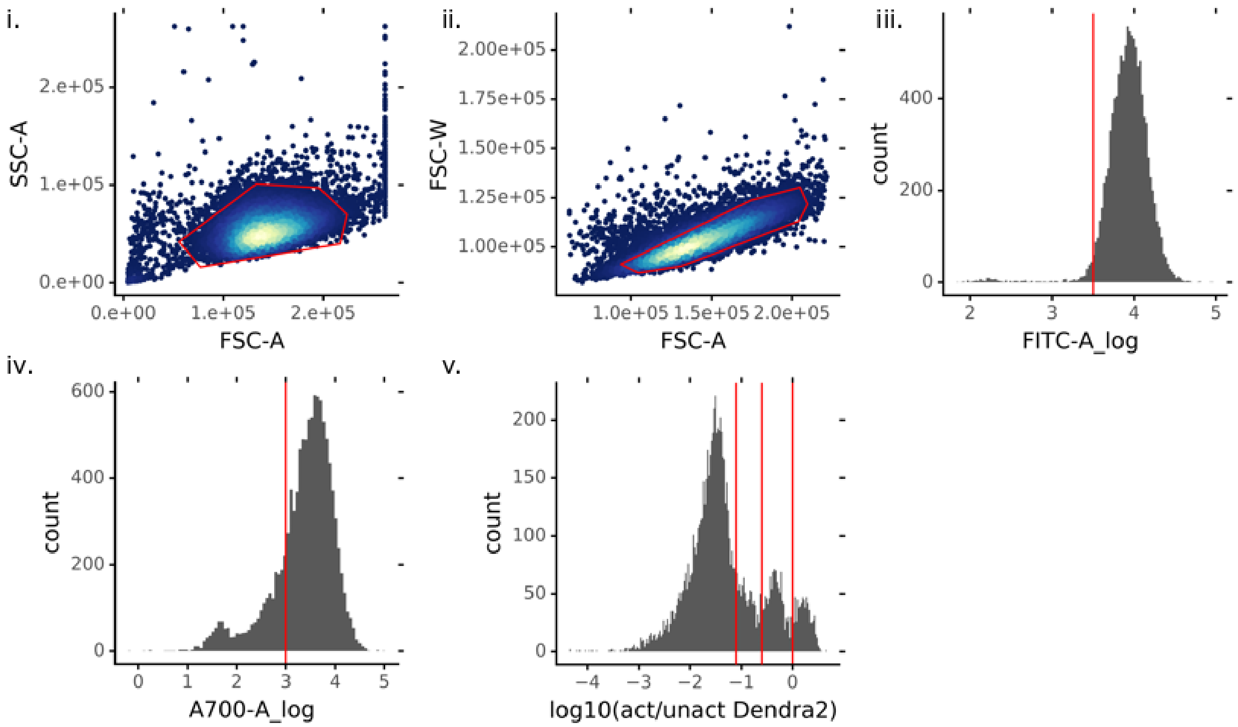


**Appendix Figure S2.** The gating scheme for Visual Cell Sorting of cells expressing the SV40 NLS library. Using the BD FACSDiva software, cells were gated as follows. **(i)** Live cells were identified using a SSC-A vs FSC-A plot. **(ii)** Single cells were gated using a FSC-W vs FSC-A plot. **(iii)** Cells expressing Dendra2 were gated using a FITC-A plot. **(iv)** Cells expressing miRFP were gated using an AlexaFluor 700-A plot. **(v)** Cells were divided into four bins according to the ratio of activated / unactivated Dendra2.

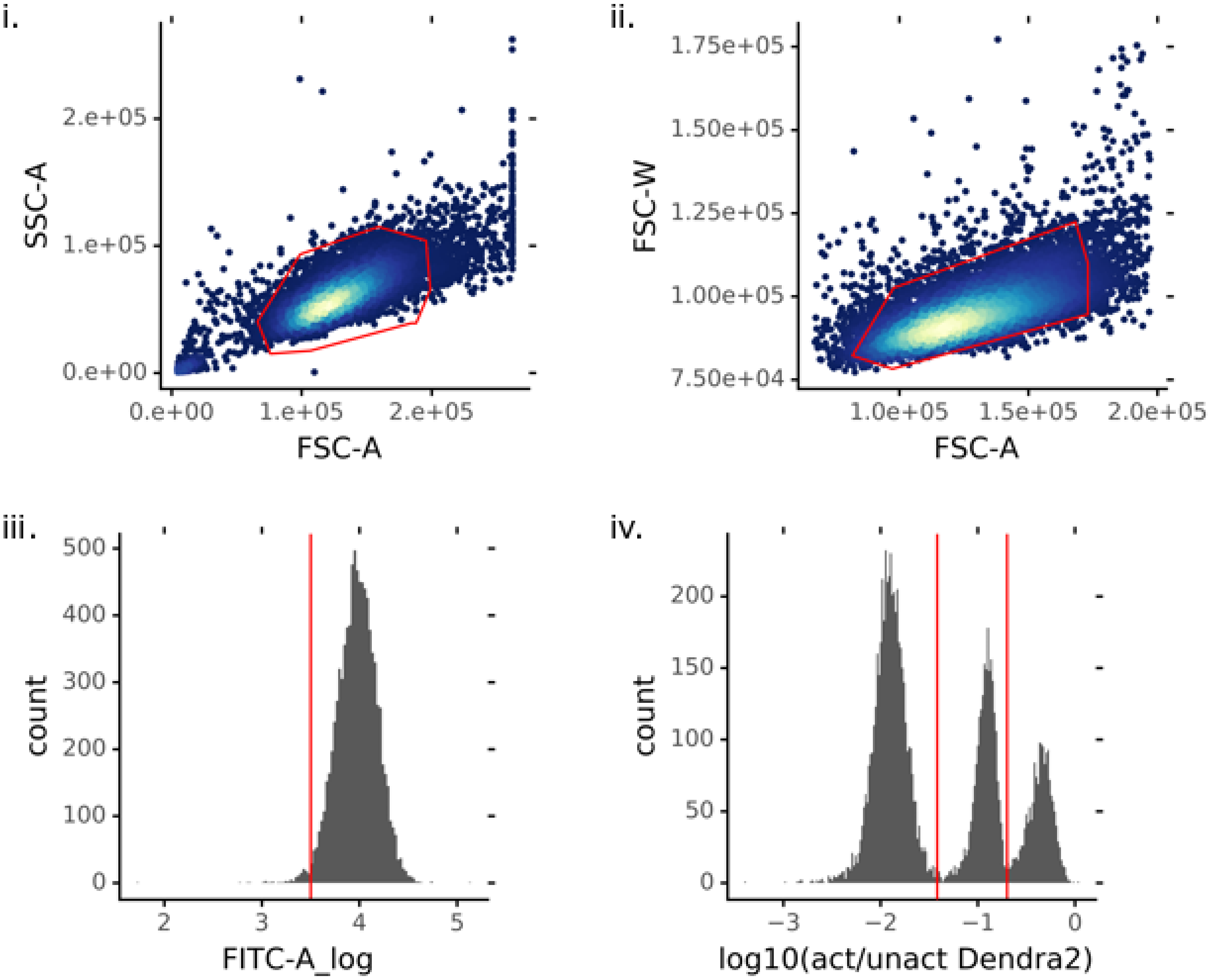


**Appendix Figure S3.** The gating scheme for Visual Cell Sorting on cells treated with paclitaxel. Using the BD FACSDiva software, cells were gated as follows. (i) Live cells were identified using a SSC-A vs FSC-A plot (ii) Single cells were gated using a FSC-W vs FSC-A plot. (iii) Cells expressing Dendra2 were gated using a FITC-A plot. (iv) Cells were divided into three bins (0 ms, 200 ms, and 800 ms) according to the ratio of activated / unactivated Dendra2. Cells in the 200 ms and 800 ms bins were sorted.
