# Supplementary material for: High‐throughput, microscope‐based sorting to dissect cellular heterogeneity
Source: Mol Syst Biol. 2020 Jun 5;16(6):e9442. doi: 10.15252/msb.20209442 (PMC7273721; doi:10.15252/msb.20209442)
Supplement: Supplementary file 2 — Expanded View Figures PDF [file MSB-16-e9442-s002.pdf]

Expanded View Figures

Figure EV1. Visual Cell Sorting.

A RPE-I cells expressing NLS-Dendra2 × 3 were imaged in the unactivated and activated Dendra2 channels; then left unactivated or activated for 50, 200, or 800 ms; and re-imaged. Scale bar = 100 µm

B U-2 OS cells expressing H3-Dendra2 under the control of a doxycycline-inducible promoter were activated with 405 nm light for 50, 200, or 800 ms; incubated for various lengths of time; and then subject to flow cytometry to determine the degree of activated Dendra2 (left panel). To examine whether shutting off Dendra2 expression before the experiment increases photoactivation ratio stability, the experiment was repeated, but doxycycline was removed from the media before cells were placed under the microscope (right panel)

C To examine the effect of Dendra2 photoactivation on cell viability, cells were activated for 800 ms, and then, apoptosis, necrosis, and death were assessed by flow cytometry using DAPI and Annexin V (n = 10,000 cells). Negative C, no photoactivation. Positive C, incubation of cells at 50 C for 10 min. The results of three independent replicates are shown.

D To test whether Dendra2 photoactivation affects gene expression, cells were activated for 800 ms, incubated for 0.5, 1.5, 2.5, 3.5, 4.5, or 6 h, and subsequently subject to bulk RNA seq. Samples were compared to two separate replicates of unactivated cells. Volcano plot of differentially expressed genes shown. Dotted line, adjusted P-value of 0.01.

© 2020 The Authors

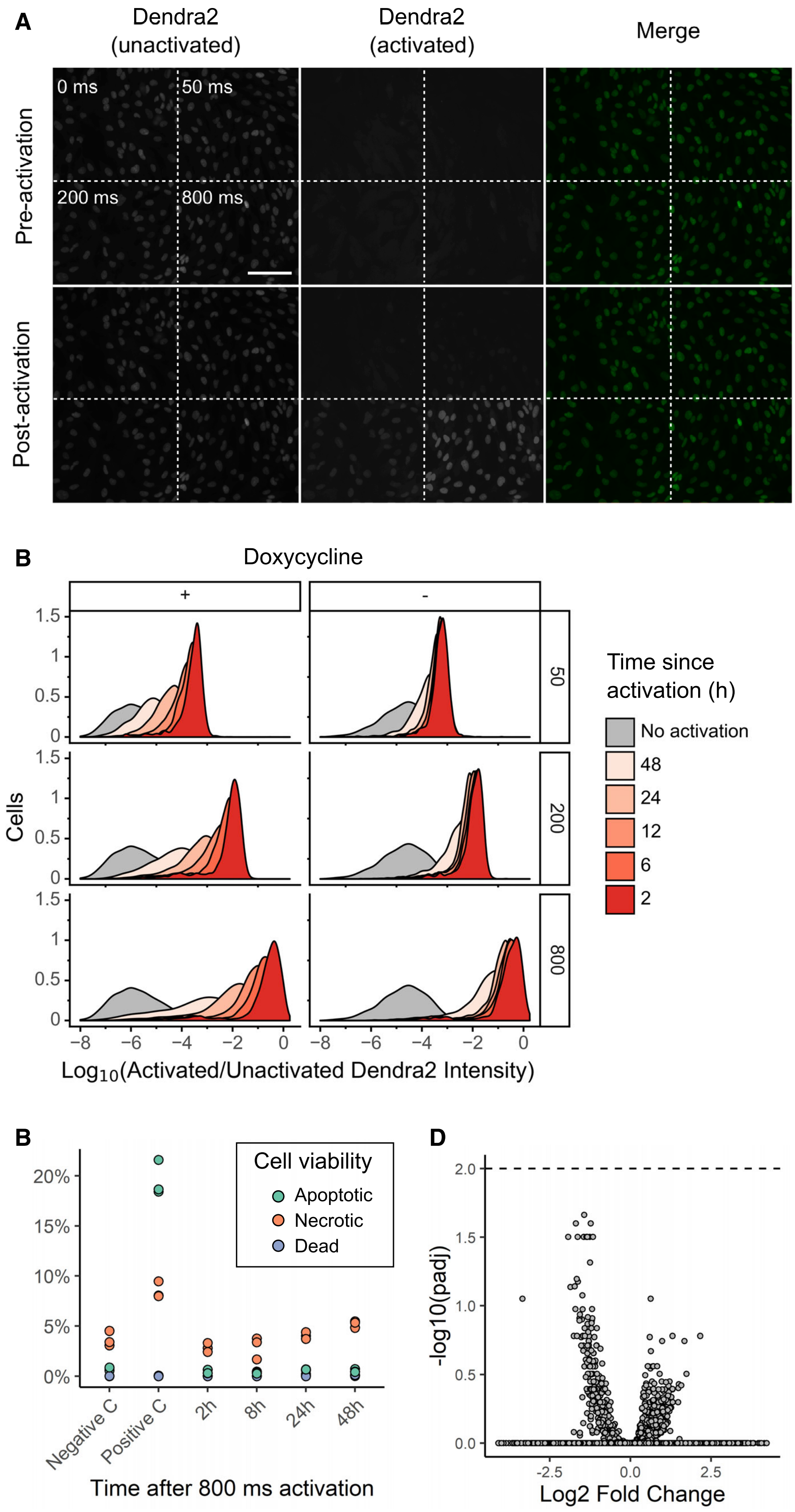

Figure EV1.

 © 2020 The Authors

Figure EV2. Visual Cell Sorting for pooled, image-based genetic screening.

A Image analysis pipeline to calculate nucleus-to-cytoplasm (NC) ratio. Nuclei were segmented using the H3-Dendra2 signal. Cytoplasmic masks were created by dilating and then removing the nuclear mask. Mean miRFP intensity was measured within each mask, and the nucleus-to-cytoplasm (N:C) ratio calculated.

B After selective photoactivation on the microscope based on N:C ratio, cells were subject to fluorescence-activated cell sorting and sorted according to their nuclear localization phenotype. Two days after sorting, cells from each sort bin were re-imaged in the miRFP channel and the nucleus-to-cytoplasm ratio reassessed (n = ~1,500 per photoactivation bin). Center line, median; box limits, 1st and 3rd quartiles; whiskers, 1.5× interquartile range; points, outliers. R1T2, recombination replicate 1, Visual Cell Sorting technical replicate 2.

C Representative images of sorted cells.

D Correlation plots of normalized scores calculated for each replicate. r^2, square of Pearson's correlation coefficient.

© 2020 The Authors

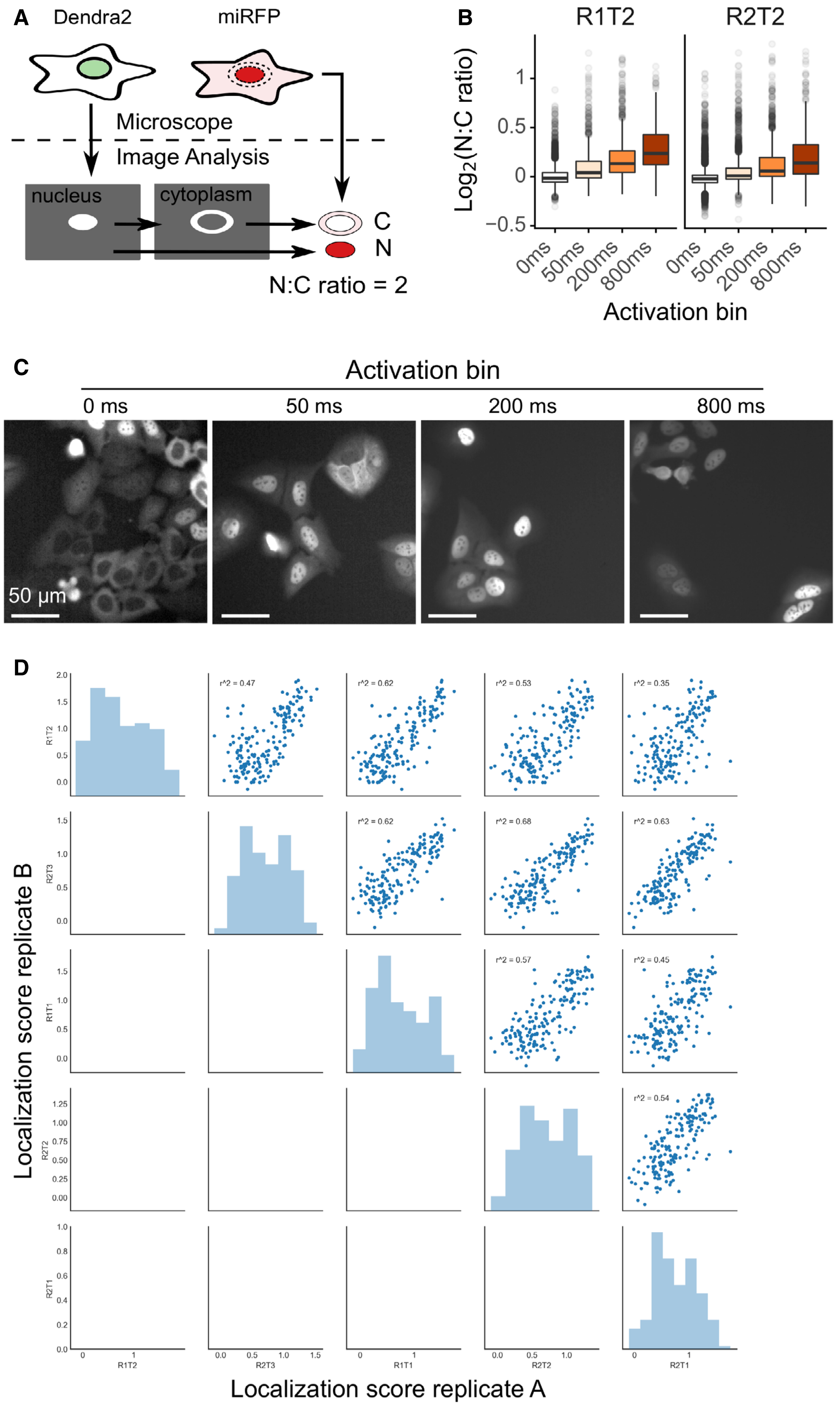

Figure EV2.

 © 2020 The Authors

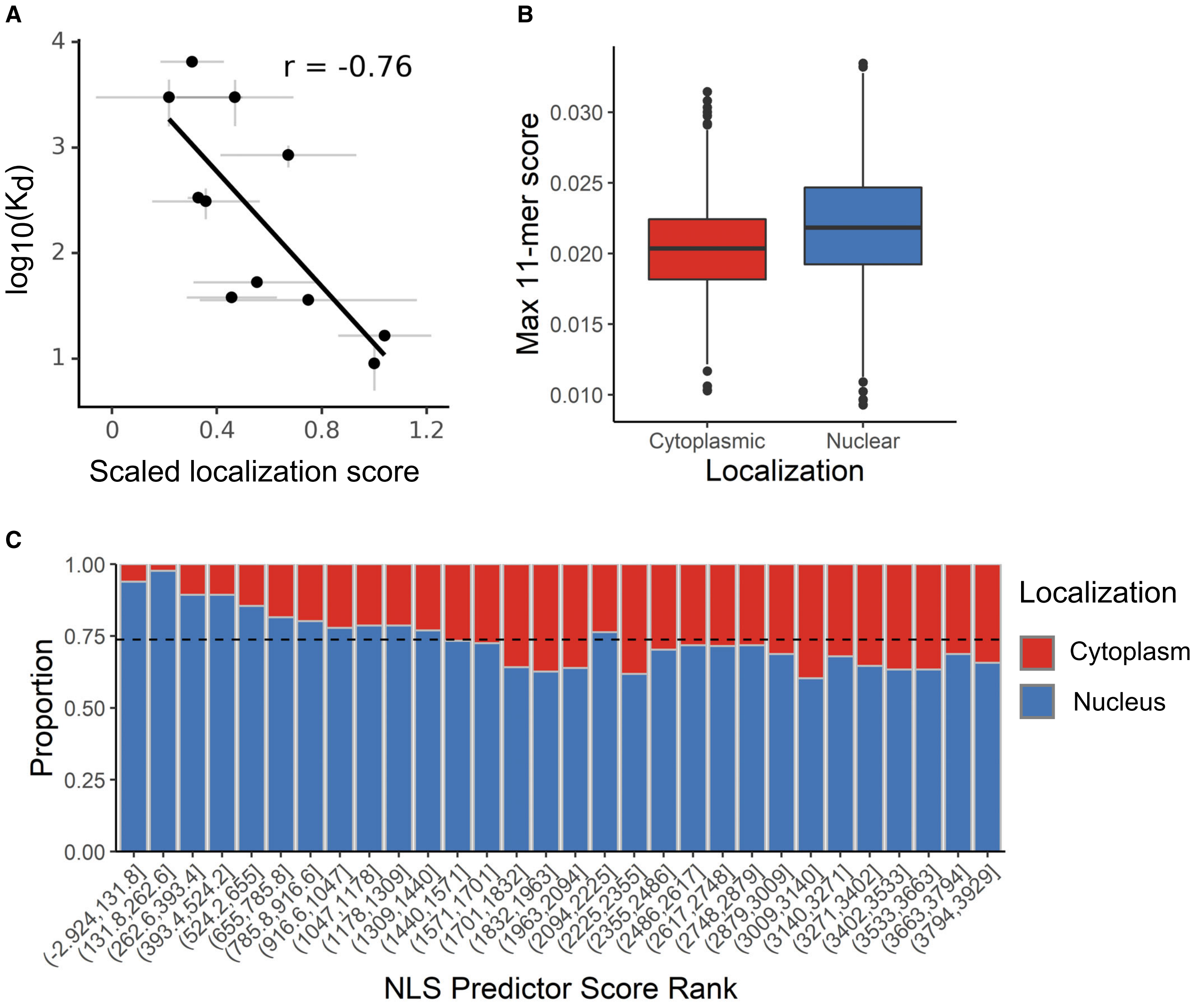

Figure EV3. Visual Cell Sorting-derived variant scores accurately predict NLS function.

A Dissociation constants measuring binding between SV40 NLS variants and importin alpha, as reported by Hodel *et al* (2001) were plotted against the variants' mean normalized scores. Gray bars, standard error from the mean. *r*, Pearson's correlation coefficient.

B All 11-mers in proteins annotated as exclusively cytoplasmic or exclusively nuclear by the Human Protein Atlas were subject to our NLS prediction model; the top-scoring 11-mer within each protein was extracted for each group (n = 3,925 11-mers; Wilcoxon rank sum P-value $< 10^{-16}$). Center line, median; box limits, 1st and 3rd quartiles; whiskers, 1.5× interquartile range; points, outliers.

C Each protein's top-scoring 11-mer was ranked and binned according to its score (n = 131 proteins per bin). Dotted line, expected proportion of nuclear proteins per bin if the model has no predictive power.

© 2020 The Authors

Figure EV4. Visual Cell Sorting to dissect heterogeneous nuclear morphology following paclitaxel treatment.

A Photoactivation gates for Visual Cell Sorting. Cells between the blue and red dotted lines represent putative normal nuclear shape factor cells activated with 405 nm light for 200 ms; and cells above the red dotted line represent putative low nuclear shape factor (lobulated) cells activated for 800 ms. Cells below the blue dotted line were not imaged or not activated

B UMAP projection of the single-cell transcriptomes derived from Visual Cell Sorting-separated lobulated and normal cells before and cell-free RNA correction with the algorithm used by SoupX (preprint: Young & Behjati, 2018)

C Visual Cell Sorting-separated cells were aligned to an unseparated, paclitaxel-treated population with the mutual nearest neighbors algorithm (Haghverdi *et al*, 2018). The first six principle components of the separated cells (*n* = 6,277), subset by nuclear phenotype and cell cycle stage, are shown. Center line, median; box limits, 1st and 3rd quartiles; whiskers, 1.5× interquartile range; points, outliers.

D Lobulation scores were generated using linear combinations of principle components 1–4. Top, Visual Cell Sorting experiment (*n* = 6,277 cells); bottom, unseparated cell population (*n* = 3,859 cells).

E Volcano plot showing DEGs significantly correlated with the lobulation score in the unseparated, paclitaxel-treated population. Red points, significant DEGs with a $\log_2$ (Effect Size), which estimates the expected $\log_2$ fold change per unit increase in lobulation score, > 0.1 and a *q*-value < 0.01.

F Raw gene counts of selected significant DEGs of cells in the unseparated, paclitaxel-treated population versus the cells' lobulation scores. Colored lines, negative binomial regression model stratified by cell cycle stage; ES, effect size, the expected fold change in gene expression per unit increase in lobulation score.

 © 2020 The Authors

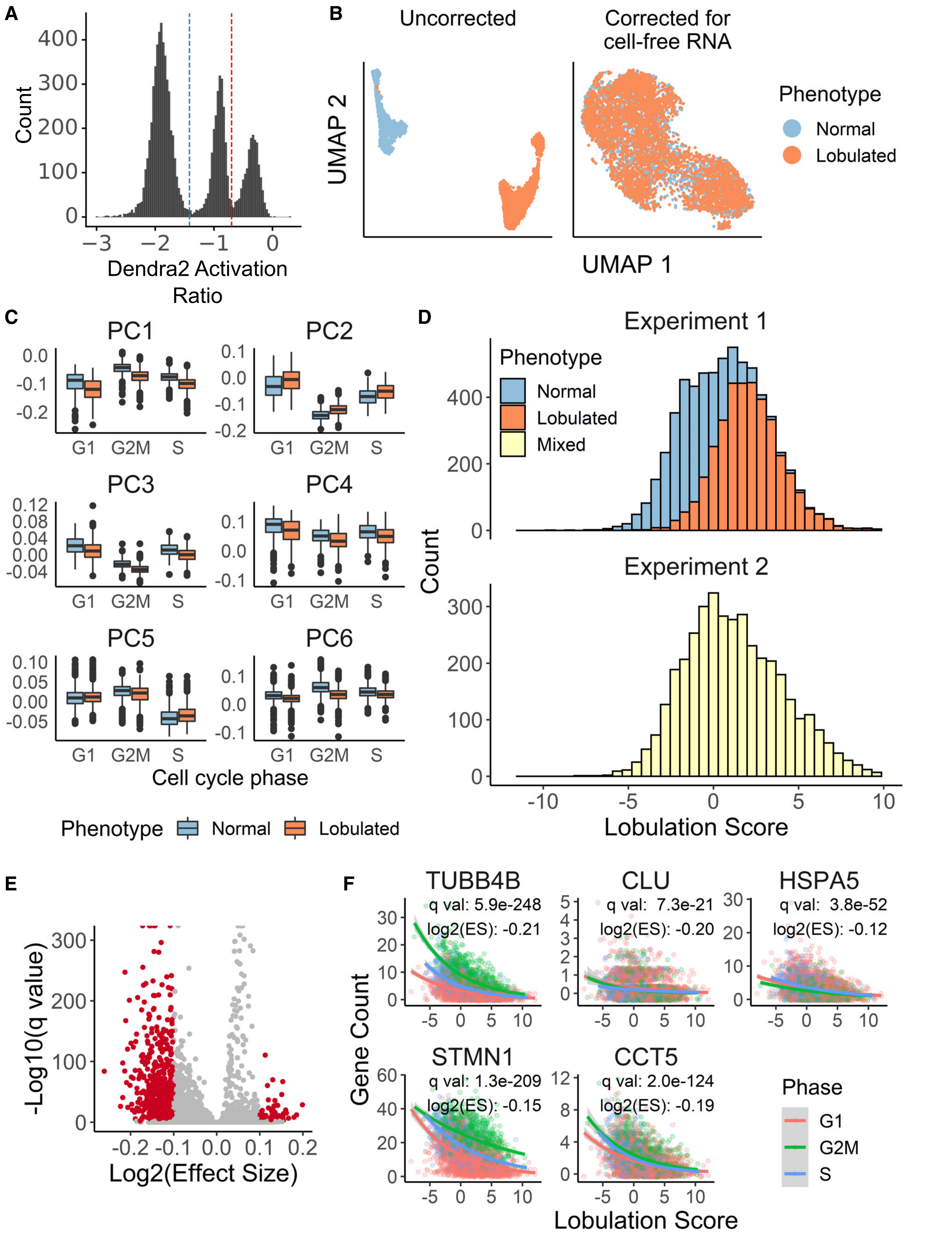

Figure EV4.

© 2020 The Authors
