# Supplementary material for: High‐throughput, microscope‐based sorting to dissect cellular heterogeneity
Source: Mol Syst Biol. 2020 Jun 5;16(6):e9442. doi: 10.15252/msb.20209442 (PMC7273721; doi:10.15252/msb.20209442)
Supplement: Supplementary file 6 — Movie EV1 [file MSB-16-e9442-s006.zip › Movie EV1 EV2 Legend.docx]

**Movie EV1, EV2.** Live cell imaging of Dendra2 at 20X magnification in asynchronous hTERT RPE-1 expressing NLS-Dendra2x3 H2B-miRFP70.3 cells treated with 0.25 nM paclitaxel for 24 hours. Each second in the videos is 1.2 hours.
